# Indwelling stents cause severe inflammation and fibrosis of the ureter via urothelial–mesenchymal transition

**DOI:** 10.1038/s41598-023-31885-1

**Published:** 2023-04-04

**Authors:** Alina Reicherz, Felipe Eltit, Kymora Scotland, Khaled Almutairi, Robert Bell, Bita Mojtahedzadeh, Michael Cox, Ben Chew, Dirk Lange

**Affiliations:** 1 Department of Urologic Sciences, The Stone Centre at Vancouver General Hospital, Jack Bell Research Centre, University of British Columbia, 2660 Oak Street, Vancouver, BC V6H 3Z6 Canada; 2 Department of Urology, Marien Hospital Herne, Ruhr-University of Bochum, Hölkeskampring 40, 44625 Herne, Germany; 3 Department of Urologic Sciences, Vancouver Prostate Centre, Jack Bell Research Centre, University of British Columbia, 2660 Oak Street, Vancouver, BC V6H 3Z6 Canada; 4 Department of Urology, David Geffen School of Medicine, University of California Los Angeles, 10833 Le Conte Avenue, Box 951738, Los Angeles, CA 90095-1738 USA; 5 College of Applied Medical Sciences, King Saud Bin Abdulaziz University for Health Sciences, C9F7+GRX, Jeddah, Saudi Arabia; 6 King Abdullah International Medical Research Center, King Abdul Aziz Medical City, C9F6+JRH, Jeddah, 22384 Saudi Arabia; 7 Department of Urologic Sciences, The Stone Centre at VGH, Jack Bell Research Centre, Room 550-3, 2660 Oak St., Vancouver, BC V6H 3Z6 Canada

**Keywords:** Urology, Ureter

## Abstract

To explore the pathways and mechanisms driving inflammation and fibrosis in stented ureters. In total, six healthy female pigs underwent cystoscopic unilateral ureteral stent insertion (6 Fr). After 14 days indwelling time, ureteral tissue was harvested in three pigs, while the remaining three pigs had their stents removed, and were recovered for 7 days. Three separate pigs served as controls. Tissue from stented and contralateral ureters was analysed histologically to evaluate tissue remodelling and classify the degree of inflammation and fibrosis, while genome, proteome and immunohistochemistry analysis was performed to assess changes at the transcriptional and translational levels. Finally, immunofluorescence was used to characterize the cell composition of the immune response and pathways involved in inflammation and fibrosis. Statistical analysis was performed using GraphPad Prism and RStudio for Welch ANOVA, Kruskal–Wallis and Dunnett’s T3 multiple comparison test. Stents cause significant inflammation and fibrosis of ureters. Gene set enrichment analysis confirmed fibrotic changes and tissue proliferation and suggests that epithelial–mesenchymal transition is a driver of fibrosis. Moreover, IL-6/JAK/STAT and TNFα via NF-κB signalling might contribute to chronic inflammation promoting a profibrotic environment. Immunostaining confirmed epithelial–mesenchymal transition in the urothelium and NF-κB expression in ureters stented for 14 days. Tissue alterations do not fully recover after 7 days. Histological evaluation showed that contralateral, unstented ureters are affected by mild inflammation. Our study showed that stenting has a significant impact on the ureter. Chronic inflammation and epithelial–mesenchymal transition are drivers of fibrosis, potentially impairing ureteral functionality in the long term. Furthermore, we observed mild inflammation in contralateral, unstented ureters.

## Introduction

Ureteral stents were first introduced by Zimskind et al. in 1967^1^. Stents are extensively used in urology to allow urine drainage in case of ureteral blocking with indications ranging from ureteral calculi to malignant obstruction, post-surgical stent insertion and other obstructing causes. As stents are associated with significant morbidity, including pain, hematuria, urgency, infections, and discomfort^2^, causing drastic effects on ureteral morphology and physiology^3^, their insertion is omitted whenever reasonable. However, as stents are often inevitable, an effort has been made to improve stent technology to prevent adverse effects^4,5^. Little progress, however, has been made to understand the mechanisms of stent-associated morbidities, long-term functional impairment and its impact on renal function^6,7^.

Clinical studies show that stents massively dilate the ureter within 5 days and cause mucosal inflammation and fibrosis^8–13^. Functional studies in stented pigs showed that stenting initially causes a hyperperistaltic phase followed by aperistalsis^14^, which has been proposed to fully recover within months^9^. We previously showed that obstruction of the ureter triggers inflammation and fibrosis of ureteral tissue^15^. Interestingly, these preliminary studies also showed increased expression of inflammatory cytokines and enzymes for tissue remodelling (TIMP-1, MMP-2, and -3) in the contralateral, unobstructed ureter, which electrophysiological studies reported is also affected by a transiently increased peristaltic rate^16^. Collectively, these data suggest the presence of unknown crosstalk mechanisms between the obstructed and unobstructed sides.

While it has been described that stenting causes inflammation and fibrotic changes in the ureter, the underlying processes are poorly understood. To address this knowledge gap, we utilized a porcine model of ureteral stenting that most closely mimics the human urinary tract and is the accepted in vivo model to study and inform clinical applicability and function of indwelling stents, to study (1) the pathways that drive inflammation in stented ureters, (2) the mechanisms of subsequent fibrosis and (3) the impact of stenting on the contralateral, non-obstructed ureter at the gene and protein levels. We confirmed fibrosis in stented ureters and showed that this may be driven by chronic inflammation involving NF-κB and the Interleukin-6/Janus kinase/signal transducers and activators of transcription (IL-6/JAK/STAT) pathway. Epithelial–mesenchymal transition (EMT) is a major driver of fibrosis in other organ systems^17–19^. Strikingly, we are the first to describe the presence of urothelial–mesenchymal transition (UMT) in stented ureters. Furthermore, we demonstrated that contralateral, non-stented ureters are mildly affected by urothelial changes, macrophage infiltration and changes in protein expression suggesting that a level of crosstalk exists between stented an unstented ureters.

Collectively, these findings expand our knowledge around the ureteral response to indwelling stents and identify pathways that drive stent-associated inflammatory responses and fibrosis, resulting in the significant morbidity experienced by patients with indwelling stents. Clinically, these pathways and mechanisms might pose therapeutic targets to minimize stent-associated symptoms and limit fibrotic changes to preserve ureteral functionality and prevent subsequent renal damage as a result of indwelling stents.

## Results

### Stent insertion damages ureteral tissue and causes inflammation

**Figure 1:**
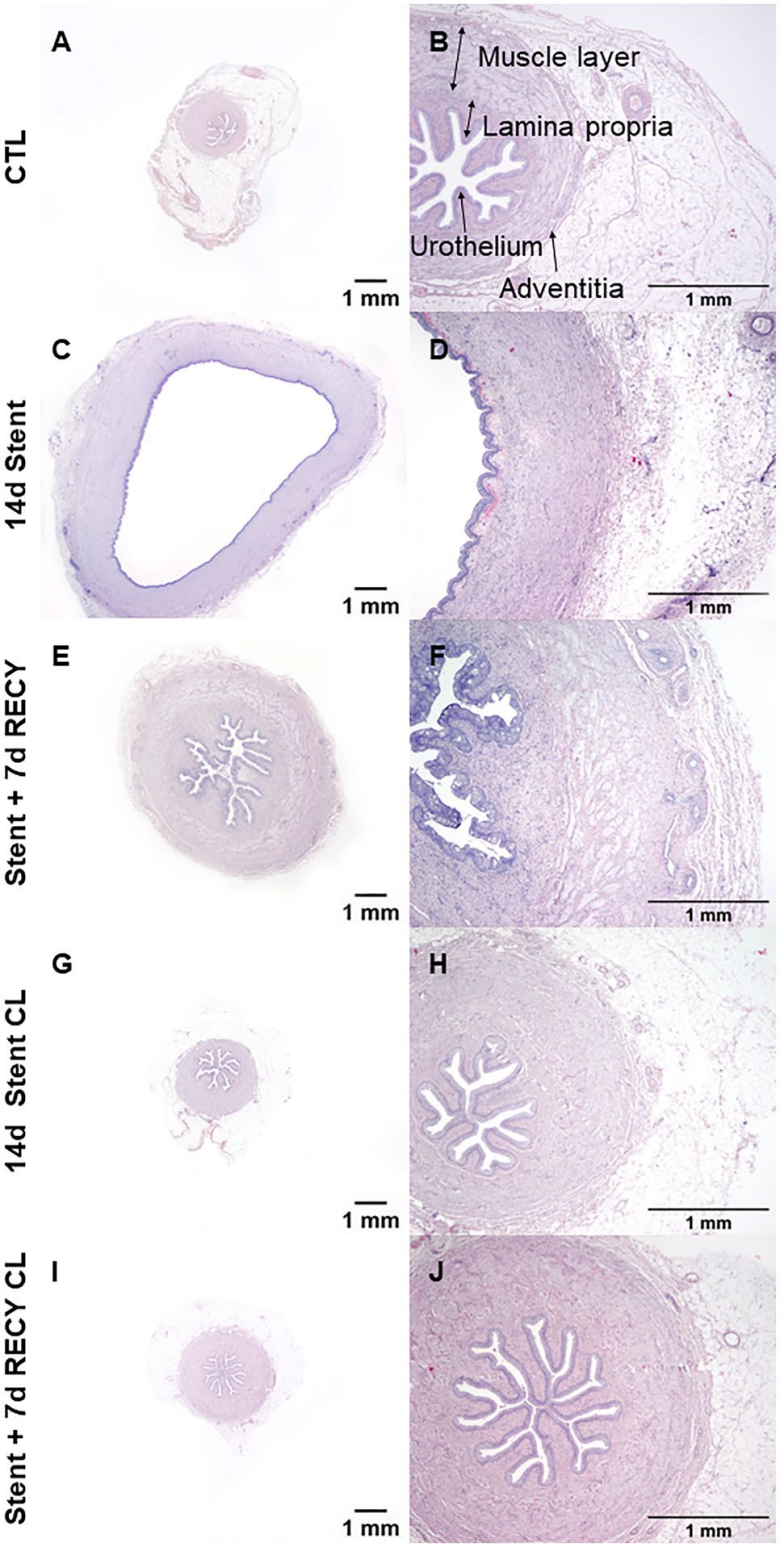
Ureteral morphology and alterations due to indwelling stents. (**A,B**) Regular morphology of control ureters (CTL). (**C,D**) Ureters stented for 14 days (14d Stent). (**E,F**) Ureters stented for 14 days, followed by stent removal and recovery for 7 days (Stent + 7d RECY). (**G,H**) Contralateral ureters of 14d Stent (14d Stent CL). (**I,J**) Contralateral ureters of Stent + 7d RECY(Stent + 7d RECYCL). Indwelling stents dilate the ureter and cause ureteral remodeling after 14 days of stent insertion. Morphology partially recovers 7 days after stent removal. Low and high magnification images of H&E stains.

Ureteral stenting causes ureteral dilatation, inflammation and fibrosis^3,5,20^. In our model, ureteral dilation increased significantly (2.2 mm to 8.1 mm) 14 days post-stent insertion driven by an increase of the lumen (0.5 mm to 4.0 mm), the muscle layer (0.3 mm to 1.6 mm) and adventitia (72 µm to 245 µm) (Table 1, Fig. 1). Seven days after stent removal the ureteral diameter partially recovered (4.3 mm) mainly due to a decrease of the lumen (1.2 mm), but also a decrease in ureteral wall thickness (1.2 mm).

**Table 1:** Histomorphological changes following ureteral stenting (µm).

	CTL, mean (SD)	14d Stent, mean (SD)	Stent + 7d RECY, mean (SD)	14d Stent CL, mean (SD)	Stent + 7d RECYCL, mean (SD)	p-value
Urothelium	47 (10)	67 (25)	47 (22)	41 (5)	44 (5)	0.73
Lamina propria	112 (8)	149 (175)	140 (92)	97 (23)	121 (31)	0.85
Muscle layer	292 (127)	1644 (769)	843 (235)	270 (16)	435 (60)	**0.03**
Adventitia	72 (25)	245 (9)	213 (151)	66 (10)	118 (35)	** < 0.0001**
Ureteral thickness	516 (107)	1766 (854)	1227 (177)	529 (37)	787 (29)	**0.003**
Ureteral lumen	480 (141)	3987 (1787)	1182 (305)	597 (146)	772 (179)	0.07
Outer ureteral diameter	2205 (368)	8077 (347)	4339 (424)	2369 (121)	2967 (48)	** < 0.0001**

Significant values are in bold.

### Indwelling stents cause an inflammatory response in stented and contralateral ureters

**Figure 2:**
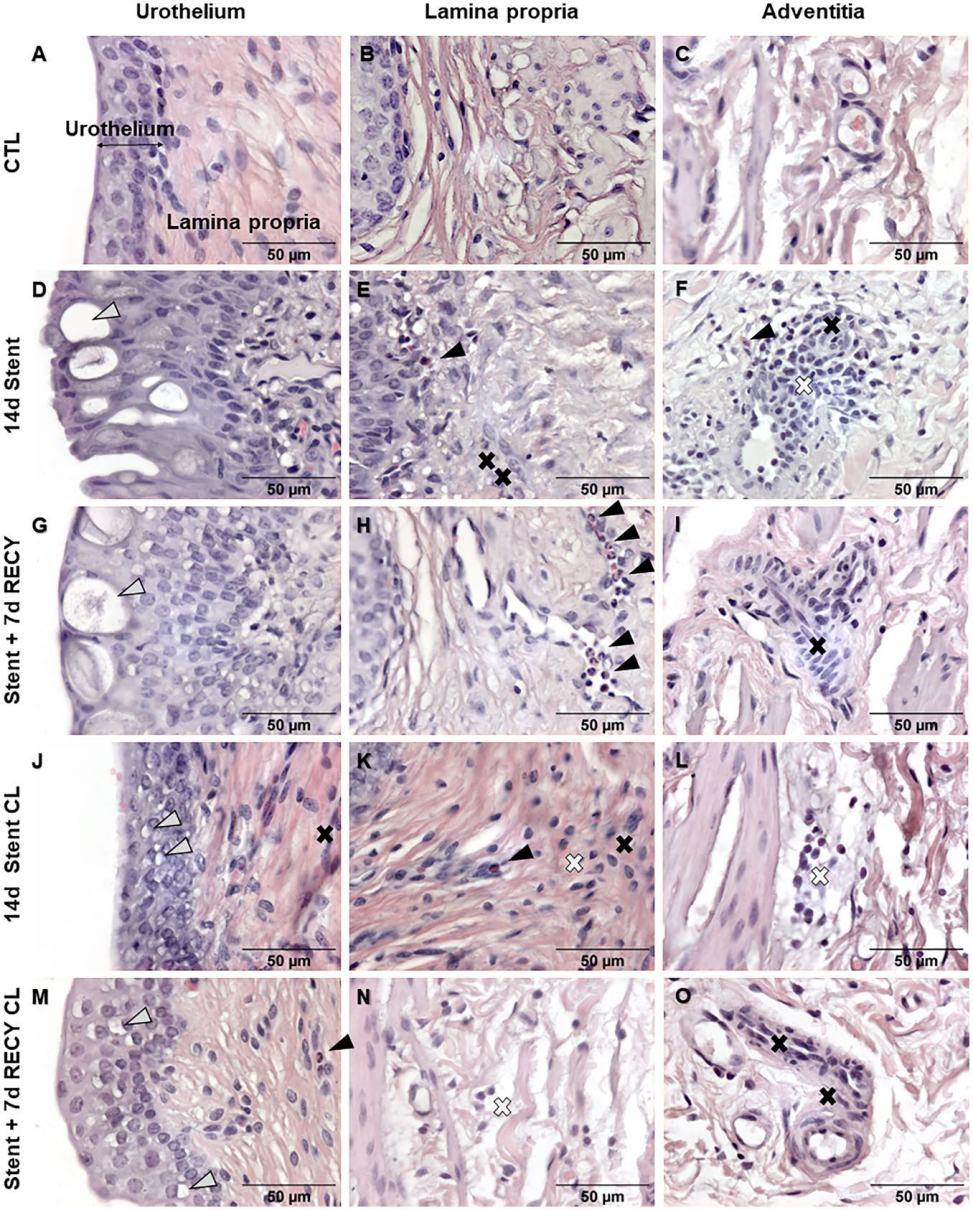
Qualitative evaluation of inflammation in stented ureters. (**A,B**) control ureter with urothelium consisting of 3–5 cell layers and an underlying lamina propria, (**C**) adventitia in a control ureter composed of loose connective tissue and normal vessel. (**D**) 14d stented ureter with thickened urothelium with 5–7 layers of cells and large vacuole (open arrowheads), (**E**) lamina propria with an increased leukocyte infiltration comprising eosinophils (arrowheads) and macrophages (black cross), (**F**) adventitia with hyperemic blood vessel transporting eosinophils (arrowheads). (**G**) 7 days after stent removal, urothelium persistently consisted of 5–7 cell layers and large vacuoles (open error head), (**H**) lamina propria with leucocyte infiltrate with eosinophils (arrowheads) and (**I**) adventitia with leukocyte infiltration and macrophages (black cross). (**J,M**) In contralateral, unstented ureters (14d Stent CL and Stent + 7d RECY), urothelium has 3–5 layers of cells with small vacuoles (open arrowheads), (**K,N**) the lamina propria is infiltrated by leucocytes comprising eosinophils (arrowhead), macrophages (black cross) and lymphocytes (white cross), and (**L,O**) the adventitia shows signs of hyperemia and macrophage infiltrates (black cross).

Previously, we described morphologic alterations in stented ureters. To further investigate the inflammatory response and cell types involved, we performed a qualitative histological analysis. Representative normal histology in unstented ureters is shown in control samples (Fig. 2A,B,C). After 14 days of stenting, we observed increased urothelial thickness, with the presence of large vacuoles (10–30 μm) and discontinuous superficial lining, although without ulceration or whole epithelial discontinuity (Fig. 2D). The lamina propria showed marked hyperaemia and leucocyte infiltration with evidence of macrophages, eosinophils and mast cells infiltrating the connective tissue (Fig. 2E). Hyperemia and diffuse infiltrates were observed in the adventitia (Fig. 2F). Large perivascular lymphocyte aggregates were observed in the stented ureter in one out of three pigs. After stent removal and 7 days recovery, the urothelium shows similar alterations, with an increased thickness and larger vacuoles, perhaps because fusion of vacuoles over time is observed closer to the luminal surface due to the natural process of urothelial desquamation (Fig. 2G). In lamina propria and adventitia, the infiltration of the macrophages, T- and B-cells was reduced but still present, and profound hyperemia was still observed (Fig. 2H,I). Remarkably, stenting affected contralateral ureters; we observed small vacuoles in the urothelium of contralateral non-stented ureters (Fig. 2J,M), clusters of mononuclear cells, predominantly macrophages, in the lamina propria (Fig. 2K), and hyperaemia in the adventitia (Fig. 2L). These changes were still present in contralateral ureters 7 days post-stent removal (Fig. 2M–O). These results demonstrate the presence of urothelial damage and inflammation in stented ureters and suggest some level of crosstalk between ureters.

Due to the relevance of macrophages and lymphocytes in inflammation and fibrosis, we studied the specific composition of the infiltrate. Macrophages are important mediators of inflammation, tissue remodelling, repair, and fibrosis. M1 macrophages initiate the first response to inflammation, while M2 macrophages have anti-inflammatory and profibrotic properties^9^. Immunofluorescence demonstrated both M1 and M2 polarized macrophages in the lamina propria and adventitia, while the lymphocyte infiltrate is composed of clusters of T- and diffuse B-cells (Fig. S2).

### Ureteral fibrosis in response to stent-induced inflammation

**Figure 3:**
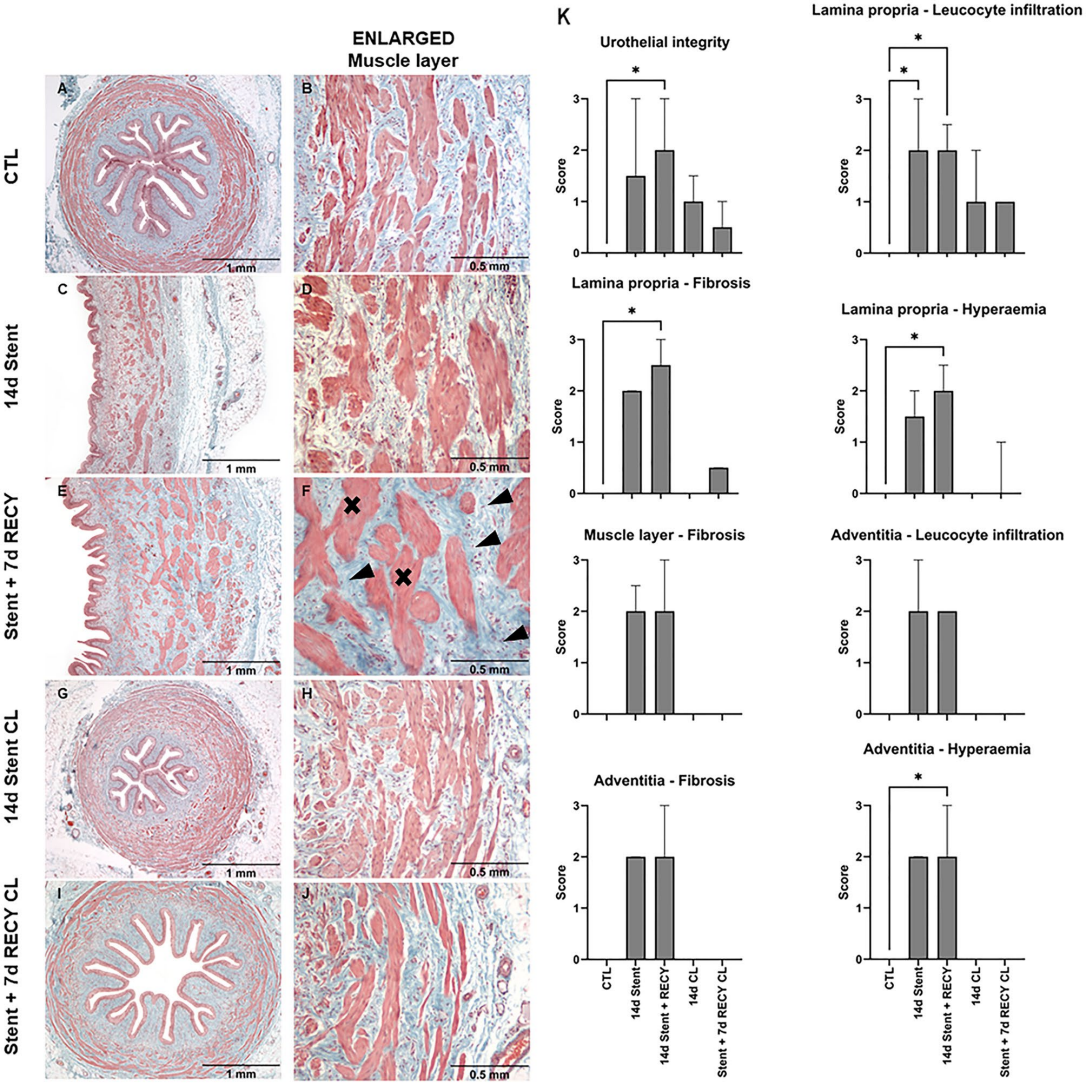
Qualitative evaluation of fibrotic changes in stented ureters (**A–J**). (**A,B**) Regular collagen distribution in control ureters (CTL), (**C,D**) ureters stented for 14 days (14d Stent) and (**E,F**) 14 days followed by stent removal and recovery for 7 days (Stent + 7d RECY) and (**G–J**) their unstented contralateral ureters (14d Stent CL and STENT + 7D RECYCL). High magnification images of the muscle layer showing extracellular matrix distribution between muscle bundles (**B**), gradual changes in 14d Stent (**D**) and a marked increase in extracellular matrix deposition (error head) and muscle hypertrophy (cross) in Stent + 7d RECY (**F**). Low and high magnification images of Goldners Trichrome. (K) Histopathological changes following ureteral stenting—median and SD. Scoring of urothelial integrity, leucocyte infiltration, hyperemia, fibrosis in the lamina propria and adventitia, and fibrosis in the muscular layer per condition. Three samples per condition were scored, and scoring was repeated two times. Kruskal–Wallis and Dunnett’s multiple comparison tests compare tissue alterations following UUO (p > 0.05 not shown).

Fibrosis is frequently non-reversible and significantly reduces organ function^21^. We previously described that complete unilateral obstruction in mice induces fibrosis^15^. Considering that stents form a partial obstruction, we investigated subsequent fibrotic changes and systematically evaluated tissue alterations in stented ureters (Fig. 3, Fig. S4). In healthy ureters, collagen fibers build a fine reticular network coating muscle cells (Fig. 3A,B). After 14 days of stenting, we observed significant collagen deposition between muscle cells (Fig. 3D). Seven days after stent removal, the muscle cells were coated with thick collagen strands (Fig. 3F). The observed changes result in fibrosis of the ureter and likely affect contractile properties. Contralateral ureters of 14d Stent (Fig. 3H) and Stent + 7d RECY animals (Fig. 3J) were not affected by increased collagen deposition.

Based on high magnification of H&E and Sirius Red stained samples, we semi-quantitatively graded the degree of urothelial integrity, fibrosis, and inflammation based on previously established parameters^15^. Quantitative results show that stents disrupt urothelial integrity and cause hyperemia and leucocyte infiltration of the lamina propria and adventitia (Fig. 3K). Furthermore, we observed fibrosis of the lamina propria, muscle layer and adventitia. Tissue alterations were still present 7 days after stent removal. The lamina propria of contralateral ureters had urothelial changes, and leukocyte infiltrates.

### Ureteral stenting induces pathways associated with inflammation and fibrosis

**Figure 4:**
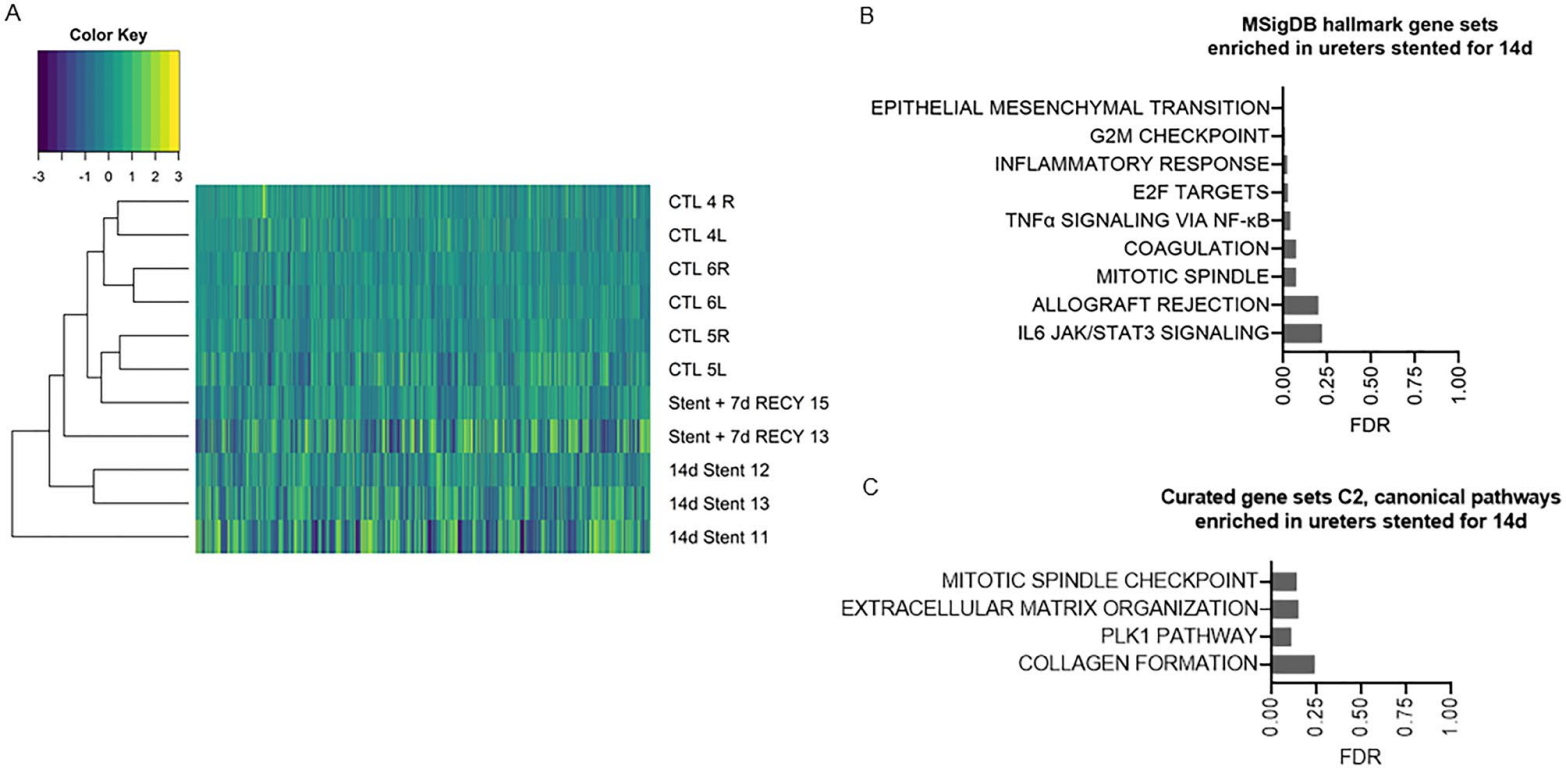
(**A**) Heatmap of gene expression of ureters stented for 14 days (14d Stent), stented for 14 days followed by 7 days of recovery (Stent + 7d RECY) and control ureters (CTL). Genes with more than 5 or more missing values were deleted (n = 10,937) (**B**) Top 9 mSIGDB Hallmark Gene Sets enriched in 14 d stented ureters with an FDR < 0.25 and p < 0.05. (**C**) Top 4 Curated gene sets canonical pathways (C2CP) enriched in 14 days stented ureters with an FDR Curated an FDR < 0.25 and p < 0.05.

We histologically described the presence of inflammation and fibrosis in stented and contralateral ureters. In order to determine if those differences are associated with specific cellular processes or a specific gene expression pattern, we performed RNAseq of homogenized tissue from control, 14d Stent, Stent + 7d RECY and contralateral ureters. By unsupervised hierarchical clustering of 14d Stent, Stent + 7d RECY and control ureters, we observed different gene expression patterns between groups, that can be segregated according to clinical conditions (Fig. 4A). Gene expression patterns of control and 14d Stent ureters differed most, while control ureters and ureters recovered after stenting were most similar (dendrogram in Fig. 4A). We did not find different expression patterns between non-stented, contralateral and control ureters (Fig. S3). Next, we performed GSEA comparing the gene expression in 14d Stent ureters and controls. GSEA of hallmark pathways revealed activation of nine pathways with a false discovery rate (FDR) below 0.25. The Hallmark EMT pathway showed the strongest enrichment, followed by G2M checkpoint, inflammatory response, E2F targets, TNFα via NF-κB, coagulation, mitotic spindle, allograft rejection and IL-6 JAK/STAT3 signalling (Fig. 4B). Subsequently, we performed a curated pathway analysis with the same criteria, which revealed the activation of four pathways: mitotic spindle checkpoint, Extracellular matrix organization, PLK1 pathway and Collagen formation (Fig. 4C).

### Urothelial–mesenchymal transition is a driver of fibrosis in chronically inflamed, stented ureters

**Figure 5:**
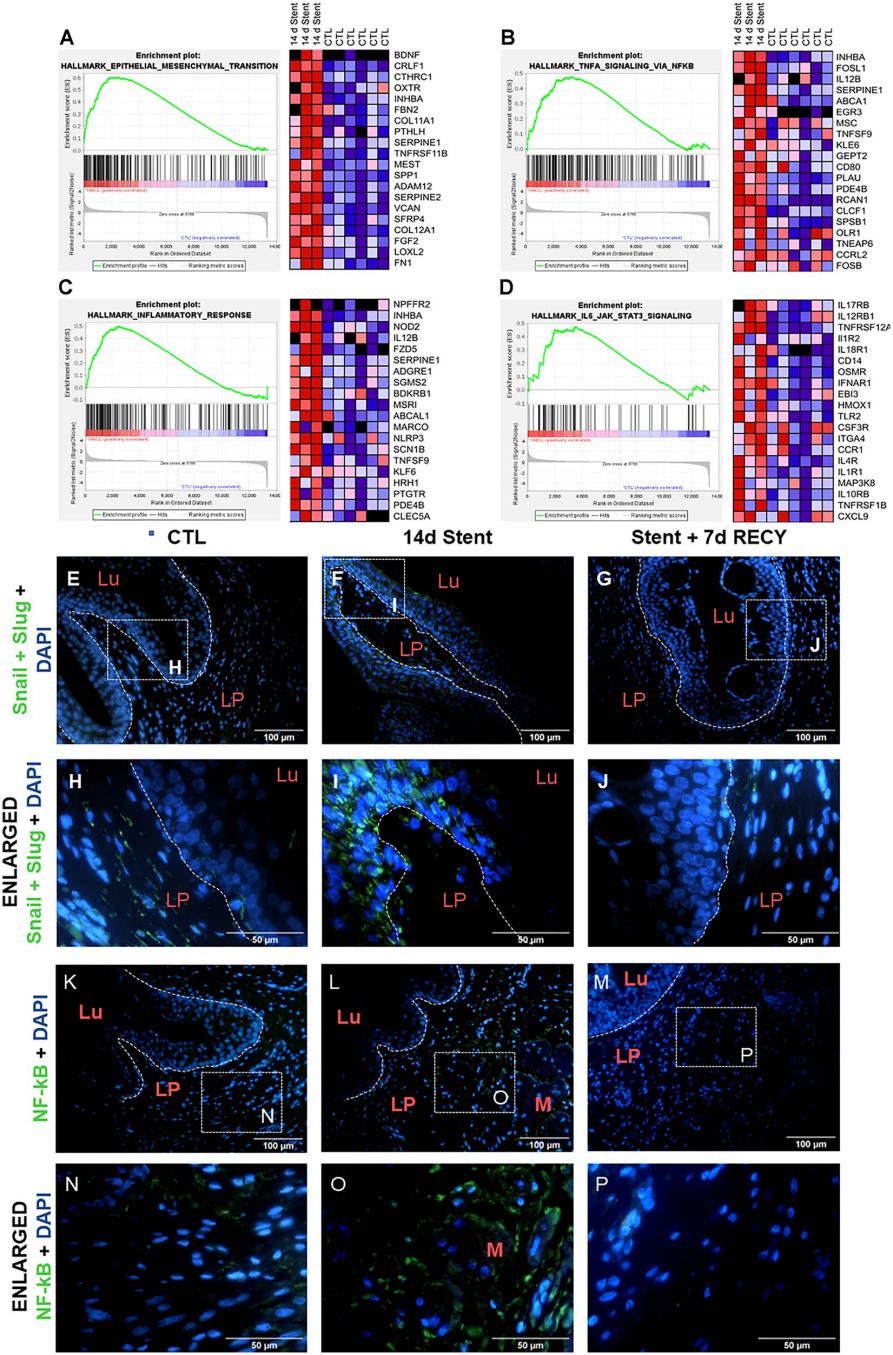
Mechanisms driving fibrosis and inflammation in stented ureters. (**A–D**) Changes in gene expression induced by 14 days of ureteral stenting compared to control ureters. (**A**) Left: Hallmark enrichment plot for epithelial–mesenchymal transition gene set; right: heat-map of top 20 genes of epithelial–mesenchymal transition gene set enriched in stented ureters. (**B**) Left: Hallmark enrichment plot for TNFα signaling via NF-κB gene set; right: heat-map of top 20 genes of TNFα signaling via NF-κB gene set enriched in stented ureters. (**C**) Left: Hallmark enrichment plot for inflammation response gene set; right: heat-map of top 20 genes of inflammation response gene set enriched in stented ureters. (**D**) Left: Hallmark enrichment plot for IL-6/JAK/STAT3 signaling gene set; right: heat-map of top 20 genes of IL-6/JAK/STAT3 signaling gene set enriched in stented ureters. (**E–P**) Immunofluorescence with transcription factors Snail + Slug and NF-kB of control (CTL), 14d stented (14d Stent), and 14d stented followed by 7d recovery (14d Stent + 7d RECY) ureters. Low and high magnification images show staining for Snail + Slug in the urothelium and NF-kB in the lamina propria and muscle layer of 14d Stent. *Lu* lumen of the ureter, *LP* lamina propria, *M* muscle layer.

Next, we analysed the genes highly enriched in the four pathways with higher enrichment: EMT (Fig. 5A), TNFα via NF-κB (Fig. 5B), inflammatory response (Fig. 5C) and IL-6 JAK/STAT3 (Fig. 5D) signalling.

GSEA analysis showed the strongest enrichment for EMT and was confirmed by enrichment of extracellular matrix organization and collagen formation. Our histomorphological evaluation showed fibrosis of the lamina propria, muscle layer and adventitia in 14d Stent and Stent + 7d RECY ureters.

Consequently, we performed immunodetection of a major transcription factor for EMT, Snail and Slug. Our results showed that in control non-obstructed ureters, there was only mild immunodetection in the lamina propria, with no detection of Snail and Slug in the urothelium (Fig. 5E,H). Interestingly a strong immunodetection was observed in the urothelium of 14d Stent ureters (Fig. 5F,I). After 7 days recovery, the ureters became negative for Snail and Slug (Fig. 5G,J), returning to their initial conditions.

### NF-κB and IL-6/JAK/STAT3 mediate inflammation in stented ureters

We previously showed M2 macrophages (Fig. S2) associated with fibrosis of stented ureters, and gene expression analysis demonstrated high enrichment of TNFα via NF-κB (Fig. 5B), inflammatory pathway (Fig. 5C) and STAT3 pathway (Fig. 5D). Thus we performed immunostaining of phosphorylated (p-65) NF-κB. Control ureters showed to be negative for phosphorylated (p-65) NF-κB (Figs. 5K,N), but found strong immunostaining in 14d Stent ureters (Figs. 5L,O). After 7 days recovery, the ureters became negative for P65 NF-κB, as the inflammatory process is decreased (Figs. 5M,P). Moreover, we performed immunostaining for STAT3 and found a nuclear activation of STAT3 in stented ureters while control ureters were negative for STAT3 (Fig. S5).

### Cell proliferation and activated fibroblasts in stented ureters

**Figure 6:**
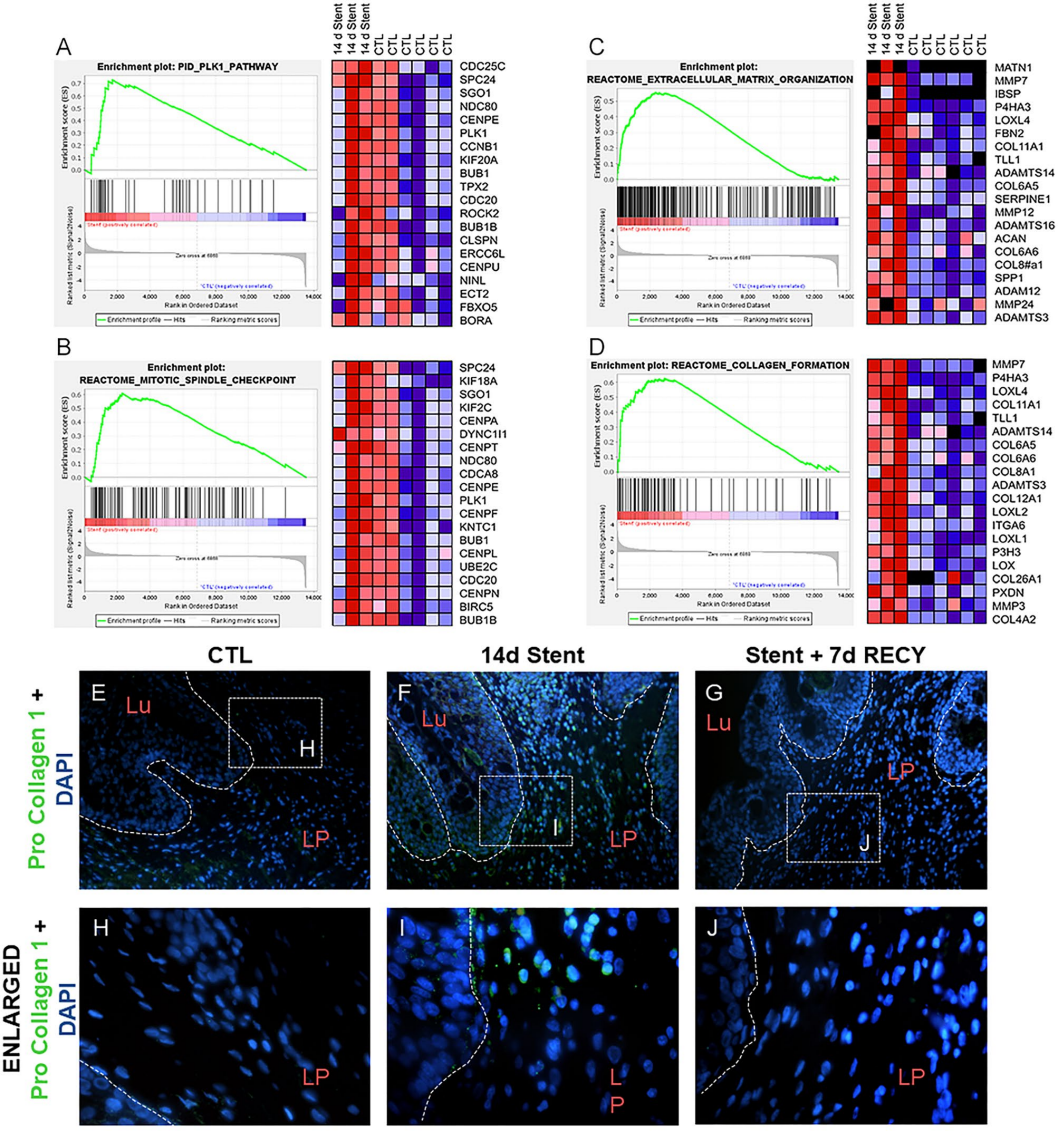
Fibrosis and cell proliferation in stented ureters. Changes in gene expression induced by 14 days of ureteral stenting compared to control ureters. (**A**) Left: curated enrichment plot for PLK-1 gene set; right: heat-map of top 20 PLK-1 gene set enriched in stented ureters. (**B**) Left: curated enrichment plot for extracellular matrix organization gene set; right: heat-map of top 20 genes extracellular matrix organization gene set enriched in stented ureters. (**C**) Left: curated enrichment plot for Reactome mitotic spindle checkpoint response gene set; right: heat-map of top 20 genes of Reactome mitotic spindle checkpoint gene set enriched in stented ureters. (**D**) Left: curated enrichment plot for Reactome collagen formation gene set; right: heat-map of top 20 genes of Reactome collagen formation signaling gene set enriched in stented ureters. (**E–J**) Immunofluorescence of control (CTL), 14d stented (14d Stent) and 14d stented followed by 7d recovery (14d Stent + 7d RECY) ureters. Low and high magnification images showing positive staining for Pro-Collagen 1 in 14d stented ureters. *Lu* lumen of the ureter, *LP* lamina propria.

The histological findings of inflammation and activation of gene sets associated with inflammation suggest that stenting elicits an inflammatory environment promoting fibrotic changes. In parallel, in the curated gene set analysis, mitotic spindle checkpoint (Fig. 6C), extracellular matrix organization (Fig. 6B), PLK-1 (Fig. 6A) and collagen formation (Fig. 6D) pathways were enriched. While mitotic spindle checkpoint and PLK-1 pathways are associated with cell proliferation, extracellular matrix organization and collagen formation directly indicate tissue fibrosis, which we described histologically in stented ureters (Fig. 3). We next performed immunodetection of Pro-Collagen 1 to determine the extracellular matrix synthesis in stented and recovered ureters. We found negative staining for Pro-Collagen 1 in non-stented ureters (Fig. 6E,H), but a strong stain in mesenchymal cells of the lamina propria in 14d stented ureters (Fig. 6F,I); the staining was negative again in ureters after 7 days of recovery (Fig. 6G,J).

### Proteomics show fibrotic remodelling of stented ureters

**Figure 7:**
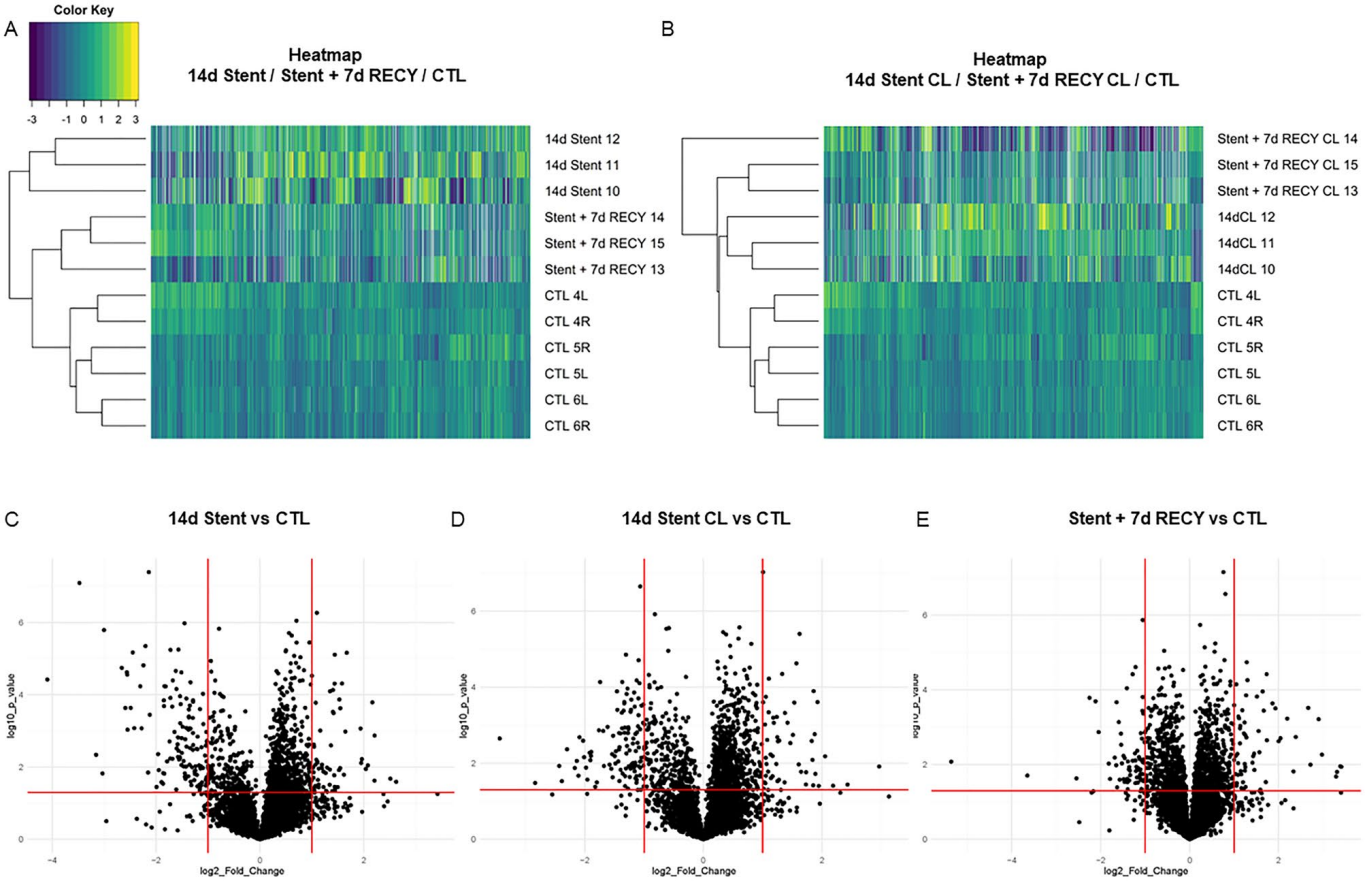
Proteomic analysis of stented, contralateral and control ureters. (**A**) Heatmap showing different protein expression patterns of ureters stented for 14 days (14d Stent), stented for 14 days followed by 7 days of recovery (Stent + 7d RECY) and control ureters (CTL). Proteins with more than 6 or more missing values were deleted (n = 5114). (**B**) Heatmap showing different protein expression of contralateral ureters from pigs stented for 14 days (14d Stent CL), stented for 14 days followed by 7 days of recovery (STENT + 7D RECYCL) and control ureters (CTL). Proteins with more than 6 or more missing values were deleted (n = 5115). (**C–E**) Volcano Plots showing proteins differentially expressed between 14d Stented vs CTL (**C**), Stent + 7d RECY and CTL (**D**), and 14d Stent CL versus CTL (**E**). Proteins with significant down- and upregulation are shown in the top left and top right quadrant. Significance was defined as p < 0.05 and at least twofold change.

Our previous results of gene expression and immunohistochemistry of cellular markers suggests that chronic inflammation triggers urothelial mesenchymal transition and increased extracellular matrix deposition in stented ureters. To evaluate weather these events are related to the histopathological features by affecting protein synthesis; we next performed a proteomic analysis of homogenized tissue from control, 14d Stent, Stent + 7d RECY and contralateral ureters. Unsupervised hierarchical clustering of proteomics showed different protein expression patterns between controls, 14d Stent and Stent + 7d RECY ureters (Figs. 7A,C,D). Interestingly, protein expression also differed between control and contralateral ureters (Fig. 7B,E). In total, 189 proteins were differentially expressed between 14 days stented and control ureters (Fig. 7C, Table S2). Among the highest fold change on upregulated proteins, we found MT3, a protein associated with regulation of redox stress in inflammation, Collagen 28α1, which is associated with extracellular matrix synthesis, Keratin 5, a cytokeratin involved in the reorganization of basal layers of epithelium after damage, and Arginase-1, a protein highly expressed in M2 macrophages, which is consistent with our immunodetection and histological observations. These results demonstrate that stenting affects protein synthesis and that some of the highly secreted proteins are involved in the previously described cellular processes.

## Discussion

In the present study, we demonstrated that ureteral stents cause inflammation and fibrosis in the affected ureter and suggest that UMT is a driver of fibrosis. Moreover, our results suggest that inflammation is mediated by TNFα via NF-κB and IL-6/JAK/STAT signalling. Interestingly, our results indicate that the inflammatory response affects the contralateral ureter. Since ureteral stents are vastly used in urology, it is important to understand the pathophysiologic response to ureteral stents and identify potential therapeutic targets to prevent associated ureteral and renal morbidities.

Our results confirm that ureteral stenting causes inflammation of ureteral tissue. In general, the inflammatory response has a protective effect; however, excessive and prolonged immune activation leads to the destruction and fibrosis of tissue. Histomorphological observation found hyperemia and leucocyte infiltration of the lamina propria, and by immunofluorescent staining, we identified M1 and M2 macrophages as well as clusters of T and B cells in stented ureters. GSEA demonstrated enrichment of inflammation-associated pathways (Hallmark inflammatory response, Hallmark TNFα via NF-kB and IL-6/JAK/STAT). Subsequently, we observed NF-kB activation by immunodetection in the lamina propria and adventitia of stented ureters. NF-κB is the most relevant transcription factor in inflammation, enhancing the transcription of inflammatory cytokines such as IL-6 and IL-8, as well as cellular mediators of inflammation^22^. Moreover, NF-kB and STAT3 are major drivers of chronic inflammation^7^. We observed STAT3 activation by immunodetection in the lamina propria of stented ureters. IL-6/JAK/STAT3 signalling induces differentiation of M2 macrophages and fibrosis^23–25^.

In line with published data, we showed that ureteral stenting causes fibrosis^12^, indicating that stents disrupt ureteral tissue and thus ureteral functionality. Our histomorphological evaluation showed fibrosis of the lamina propria, muscle layer and adventitia, which was confirmed by GSEA that showed enrichment of extracellular matrix organization and collagen formation gene sets. Further we observed by Immunofluorescence the presence of activated fibroblasts (Pro-Collagen 1) in the lamina propria of stented ureters. Organ fibrosis results from extracellular matrix deposition by activated fibroblasts. ‘EMT’ refers to the transition of epithelial cells to cells with a mesenchymal phenotype, capable of secreting extracellular matrix components^17,19^. EMT is an important source of fibroblasts during fibrosis. In renal fibrogenesis, 36% of fibroblasts derive from local EMT^19^. GSEA demonstrated strong enrichment of the EMT gene set, and immunostaining with Snail + Slug confirmed activation of the major transcription factor for EMT^18^. EMT progression is mediated by inflammation^17,19^: the Hedgehog pathway is involved in EMT regulation. Our workgroup has previously described that stenting causes a downregulation of the Hedgehog Effector Gli1 in stented smooth muscle cells^3^. Furthermore, IL-6, which has been reported to promote EMT via JAK/STAT3^24^, was upregulated in GSEA in stented ureters. These results demonstrate the role of EMT/UMT in fibrosis of stented ureters.

Our study shows that stenting has a mild effect on the contralateral ureter. While we found no difference in the thickness of ureteral layers, we observed urothelial changes and clusters of macrophages infiltrating the lamina propria of contralateral ureters. GESA of Hallmark genes sets did not show enrichments; however, unsupervised hierarchical clustering showed a difference in protein expression between control and contralateral ureters. These findings contribute to recent results from our workgroup, showing an increase of inflammatory cytokines, TIMP-1 and matrix-metalloproteases in the contralateral ureter compared to control ureters in a mouse model of unilateral ureteral obstruction^15^. Hammad et al. previously reported that complete and partial ureteral obstruction affected the peristaltic frequency of the contralateral ureter and hypothesized that humoral or neural factors might be involved^16^. Our results suggest that contralateral ureters are affected by ureteral stenting through unknown mechanisms possibly involving a systemic inflammatory response, which we previously hypothesized^26^. The clinical impact on the contralateral ureter, however, is uncertain.

While our findings on inflammatory pathways and UMT in stented ureters are novel, we acknowledge that our study has some limitations. While the findings are based on samples from only n = 3 animals, our previous studies have found this to be sufficient to obtain statistically significant differences given the striking changes in ureteral structure and physiology induced by stenting. As the specimen from one pig in the Stent + 7d RECY group was not eligible for genome analysis, the power of analysis for reversibility of changes may be limited, although it must be noted that statistically significant differences were still obtained. The antibodies used are not specific to the porcine species. Nevertheless, we believe that identifying drivers for inflammation and fibrosis contributes to the basic understanding of the pathophysiologic response to stenting. Since ureteral stenting leads to significant patient morbidity, the identified pathways might pose new therapeutic targets.

The present study shows that stent insertion severely impacts the ureter, causing acute and chronic inflammatory responses eliciting UMT, a major driver of ureteral fibrosis.

## Methods

The methods are reported in accordance with ARRIVE Guidelines.

### Ureteral stenting in pigs

All procedures involving animals were approved by the Animal Care Committee of the University of British Columbia (A18-0251) and were carried out in accordance with relevant guidelines and regulations**.** For the porcine model of ureteral stenting, only Female Yorkshire Farm Pigs (30 kg) were used, as the male urethral anatomy (spiral) makes it difficult to insert a stent without tissue damage that will affect relevant analyses carried out in this study. The present study includes tissues from animals divided into two groups (n = 3 female pigs/group). Based on our previous experience using this porcine model of ureteral stenting, a sample size of n = 3 animals is sufficient to detect statistically significant differences in ureteral physiology and function. Animals in both groups randomly had a stent inserted either in the right or left ureter which remained indwelling for 14 days. Animals in the 14d Stent group underwent stent removal and tissue collection on day 14, while animals in Stent + 7d RECY group underwent stent removal on day 14 and tissue collection following a 7-day recovery period. Three additional pigs served as controls that did not undergo any procedure (CTL). Overall, analyses were carried out on n = 3 14 days stented and n = 3 corresponding contralateral unstented ureters, n = 3 14d stented and recovered ureters and n = 3 corresponding contralateral ureters, and a total of n = 6 ureters for the control group. Results from all animals were included in the analysis. Stents (6 Fr, Boston Scientific) were inserted cystoscopically under general anaesthesia using isoflurane. Euthanasia was performed using intravenous administration of sodium pentobarbital. Ureteral tissue proximal to the obstruction and the contralateral ureter were collected for further analysis. Procedures have been described previously; Peristalsis was observed visually over a two-minute interval following open laparotomy prior to euthanasia as previously described^3,27^.

### Histology and histopathological evaluation

Ureters were fixed in 10% buffered formalin for 48 h, dehydrated and paraffin embedded. Five-micron sections were stained with hematoxylin and eosin (H&E), Picro-Sirius red and Goldner's Trichrome, which are standard stains to evaluate morphology and collagen alignment^28^. The slides were photographed under light microscopy and polarized light using a cooled camera and ZEN software (Carl Zeiss Canada). Evaluation of slides comprised measurements of histologic dimensions (thickness of the ureter, urothelium, lamina propria, muscle layer and adventitia, outer diameter and lumen) (Fig. S1) and histopathological grading of slides from every condition^15^*.* We used ImageJ 1.53e software for digital image analysis. Histopathological grading was performed according to an established scoring system for inflammation and fibrosis by FE, a researcher experienced in histopathology, blinded to the sample's condition (Table S1).

### Immunofluorescence

To retrieve antigens, histology samples were incubated in citrate buffer (pH  6.0) at 95 °C for 40 min prior to unspecific blocking with 3% BSA in PBS for 20 min. The slides were incubated overnight with primary antibodies (diluted 1:100) at 4 °C, washed, and incubated with secondary antibodies (diluted to 1:100) for 90 min at 37 °C. The list of utilized antibodies is shown in Table 2. Nuclei were counterstained with Hoechst 33342, Trihydrochloride, Trihydrate (H3570; ThermoFisher Scientific), dehydrated in graded alcohol, cleared in xylene and mounted using an antifading mounting medium (S3023; Dako). Imaging was performed as described above.

**Table 2:** Primary and secondary antibodies utilized for immunofluorescence.

	Host species	Code	Supplier
Primary antibodies			
Pro-collagen 1 antibody	Rabbit	bs-0578R	BIOSS
CD3e antibody	Mouse	MA1-7630	ThermoFisher Scientific
Anti-CD20 antibody	Rabbit	ab64088	Abcam
Anti-CD 163 antibody	Rabbit	ab182422	Abcam
Anti-CD 68 antibody	Mouse	968-MSM2-P1	ThermoFisher Scientific
NF-kB antibody	Rabbit	ab32536	Abcam
Sail + Slug antibody	Rabbit	ab85936	Abcam
Anti-S**TAT3**	Rabbit	ab32143	Abcam
Secondary antibodies, fluorescently labelled			
F(ab')2-Goat anti-Rabbit IgG (H + L)	Donkey	A-11070	ThermoFisher Scientific
Goat anti-Mouse IgG (H + L)	Donkey	A-11005	ThermoFisher Scientific

### Genomic analysis

RNAseq was performed on porcine ureteral tissue stented for 14 days (n = 3), Stent + 7d RECY (n = 2) and controls. For total RNA extraction, fresh frozen tissue was homogenized, and RNA was extracted using the Maxwell RSC Simply RNA Tissue kit (Promega Corporation, Madison, WI), according to the manufacturer's instructions. RNA quality was assessed by TapeStation (Agilent Scientific Instruments, Santa Clara, CA) and Qubit Fluorometric quantitation (ThermoFisher Scientific, Waltham, MA). We prepared RNA libraries using TruSeq Stranded mRNA Library Prep Kit (Illumina Inc., San Diego, CA) and assessed their quality by TapeStation, Qubit and qPCR quantitation. Libraries were pooled and sequenced on the NextSeq 500 (Illumina Inc., San Diego, CA), aiming for at least 20 M reads per library.

### Proteomic analysis

For protein collection, approximately 100 mg of ureteral tissue was homogenized and centrifuged. Peptide concentration was determined via Nanodrop spectrophotometer (ThermoFisher Scientific, Waltham, MA). Next, 35 µg of each sample was used for subsequent 10plex TMT labelling. Additionally, a pooled sample containing 4 µg of each sample was prepared. Approximately 120 µg of each combined sample underwent high pH fractionation using a Waters Acquity UPLC coupled with an Acquity Photodiode Array detector.

For data acquisition (control software version 3.1.2412.25), a data-dependent method with Synchronous Precursor Detection (SPS) was utilized. Data files were processed with Protein Discoverer 2.2.0.388 (ThermoFisher Scientific, Waltham, MA) and searches were carried out using Sequest HT against the Sus Scrofa SwissProt TaxID = 9823 (v2017-10-25) database with trypsin cleavage, maximum missed cleavage 2, minimum peptide length 5, precursor mass tolerance 10 ppm, fragment mass tolerance 0.6 Da with K,M,P oxidation and K TMT6plex as permitted peptide dynamic modifications, N terminal acetyl as dynamic protein modification, and with static carbamidomethyl C and peptide N terminus TMT6plex modifications. Sample arms were grouped into proximal ureters from 14d Stent versus Stent + 7d RECY along with corresponding controls for each group.

### Statistical analysis

Statistical analysis was performed using GraphPad Prism 9.2 software (GraphPad Software, United States) and RStudio 2021.09.0. Welch ANOVA test was used to compare the thickness of ureteral layers, and Kruskal–Wallis and Dunn's T3 multiple comparisons test to compare histological alterations and immunoassay concentrations. Differences were considered statistically significant for p < 0.05. We determined changes in gene expression of specific pathways in ureters stented for 14 days/contralateral ureters of stented pigs compared to control ureters using Gene Set Enrichment Analysis (GSEA)^29,30^. First, a hallmark gene set analysis (MsigDB version 7.5) was performed. Subsequently, we performed a curated pathway analysis (C2CP).

## Supplementary Information

Supplementary Information.

## Data Availability

The datasets generated and/or analysed during the current study are available in the following repositories: (1) The mass spectrometry proteomics data have been deposited to the ProteomeXchange Consortium via the PRIDE partner repository (https://www.ebi.ac.uk/pride/) with the dataset identifier PXD038161. Reviewer account details: Username: reviewer_pxd038161@ebi.ac.uk. Password: YPbBe8Er. (2) The RNAseq dataet has been uploaded to the European Genome-Phenome Archive (EGA) and is accessible under the following link: https://ega-archive.org/studies/EGAS00001006855. Request for data access will be referred directly to the EGA Data Access Committee: https://ega-archive.org/dacs/EGAC00001002997. For data requests please contact the corresponding author Dr. Dirk Lange (dirk.lange@ubc.ca).

## Abbreviations

CTL: Control
EMT: Epithelial–mesenchymal transition
H&E: Hematoxylin and eosin
JAK: Janus kinase
NF-κB: Nuclear factor kappa-light-chain-enhancer of activated B cells
STAT: Signal transducers and activators of transcription
TGF-ß: Transforming growth factor-beta
UMT: Urothelial–mesenchymal transition
14d Stent: Ureters stented for 14 days
14d Stent + 7d RECY: Ureters stented for 14 days followed by stent removal and recovery for 7 days
14d Stent CL: Unstented, contralateral ureter of a pig that was stented for 14 days
Stent + 7d RECYCL: Unstented, contralateral ureter of a pig that was stented for 14 days followed by stent removal and recovery for 7 days
