# Supplementary material for: Indwelling stents cause severe inflammation and fibrosis of the ureter via urothelial–mesenchymal transition
Source: Sci Rep. 2023 Apr 4;13:5492. doi: 10.1038/s41598-023-31885-1 (PMC10073185; doi:10.1038/s41598-023-31885-1)
Supplement: Supplementary file 1 — Supplementary Information. [file 41598_2023_31885_MOESM1_ESM.pdf]

Supplements to

Indwelling Stents Cause Severe Inflammation and Fibrosis of the Ureter via Urothelial-Mesenchymal Transition

Alina Reicherz[1,2,], Felipe Eltit[1,3], Kymora Scotland[4], Khaled Almutairi[1], Robert Bell[3], Bita Mojtahedzadeh[3], Michael Cox[3], Ben Chew[1], Dirk Lange[1,*]

1 The Stone Centre at Vancouver General Hospital, Department of Urologic Sciences, University of British Columbia, Vancouver, BC, Canada.

2 Department of Urology, Marien Hospital Herne, Ruhr-University of Bochum, Herne, Germany

3 Vancouver Prostate Centre, Vancouver, BC, Canada

4 Department of Urology, UCLA David Geffen School of Medicine, Los Angeles, USA

5 College of Applied Medical Sciences, King Saud Bin Abdulaziz University for Health Sciences, Jeddah, Saudi Arabia

6 King Abdullah International Medical Research Center, Jeddah, Saudi Arabia

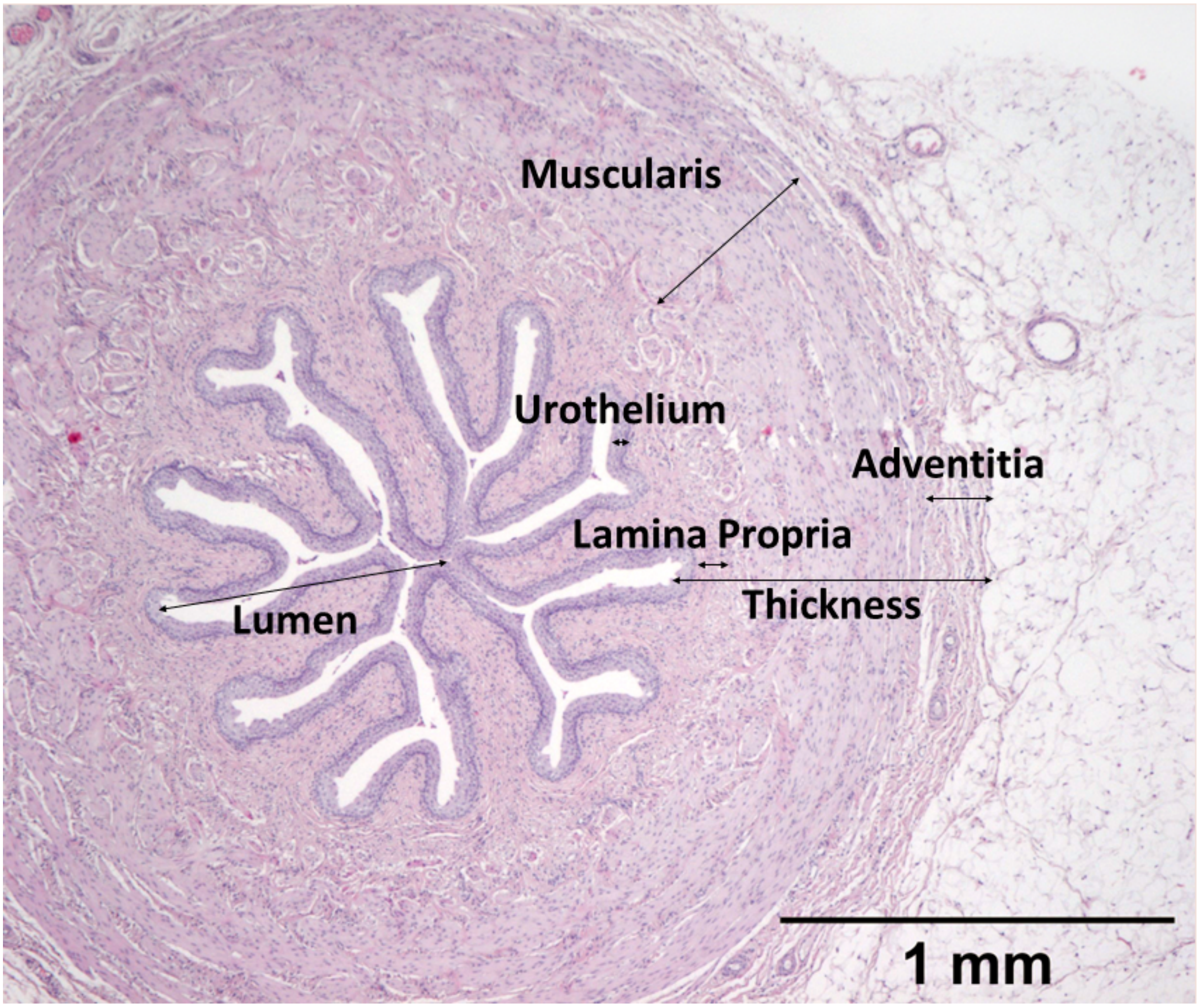

Figure S 1: Histological evaluation: Measurements of histologic dimensions (thickness of the ureter, urothelium, lamina propria, muscle layer and adventitia, diameter and lumen)

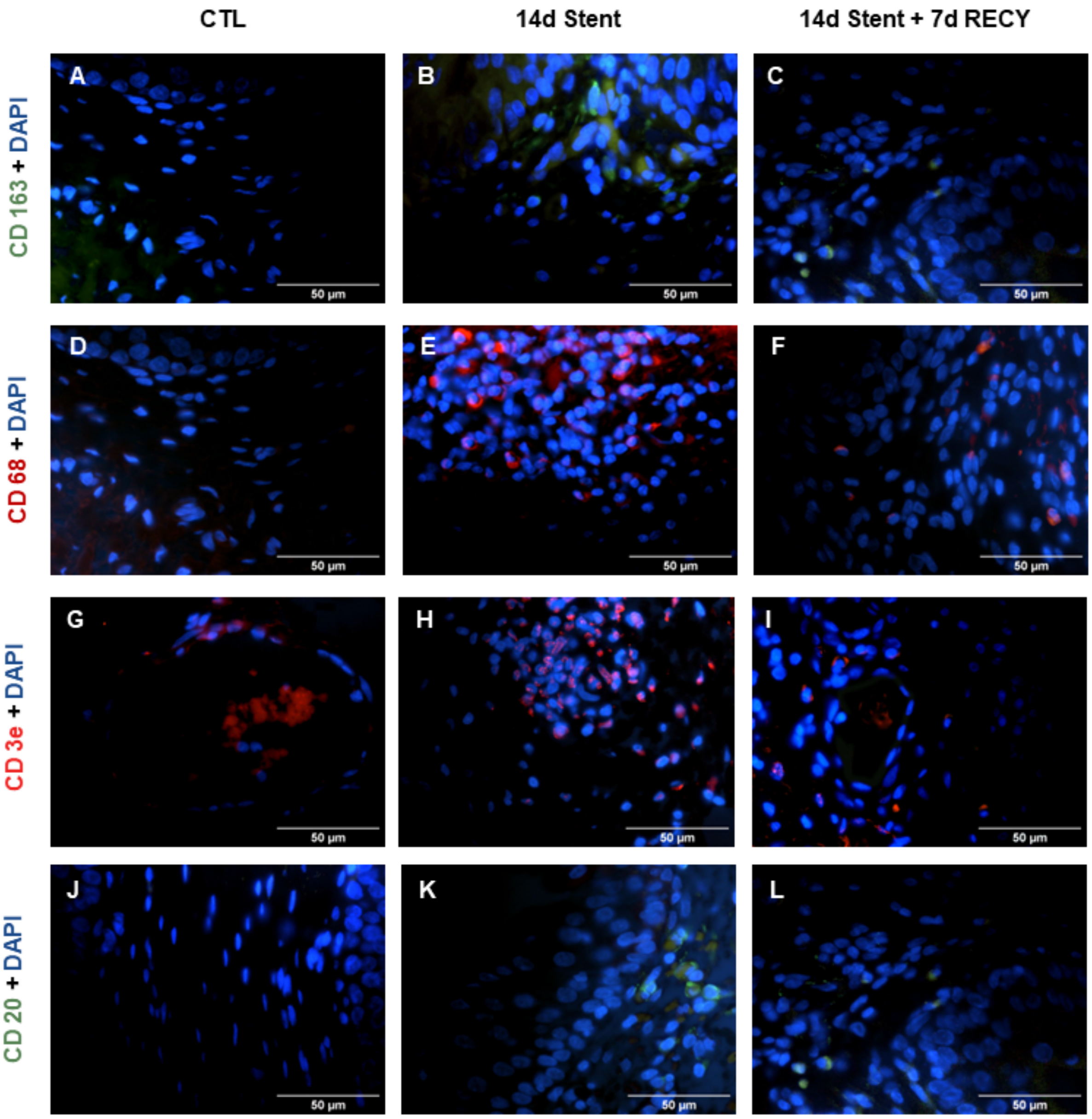

Figure S 2: Immunofluorescent staining of leucocyte subtypes in ureters. High magnification images of the showing staining for M1 macrophages (CD163), M2 macrophages (CD 68), T-cells (CD3e) and B-cells (CD20) in ureters stented for 14 days (14d Stent), stented for 14 days followed by stent removal and a 7-day recovery (Stent + 7d RECY) and control ureters (CTL). In 14d Stent ureters clusters of T-cells are present, and M1 and M2 macrophages and B-cells diffusely infiltrate the tissue. Leucocyte infiltration partially resolves during the recovery.

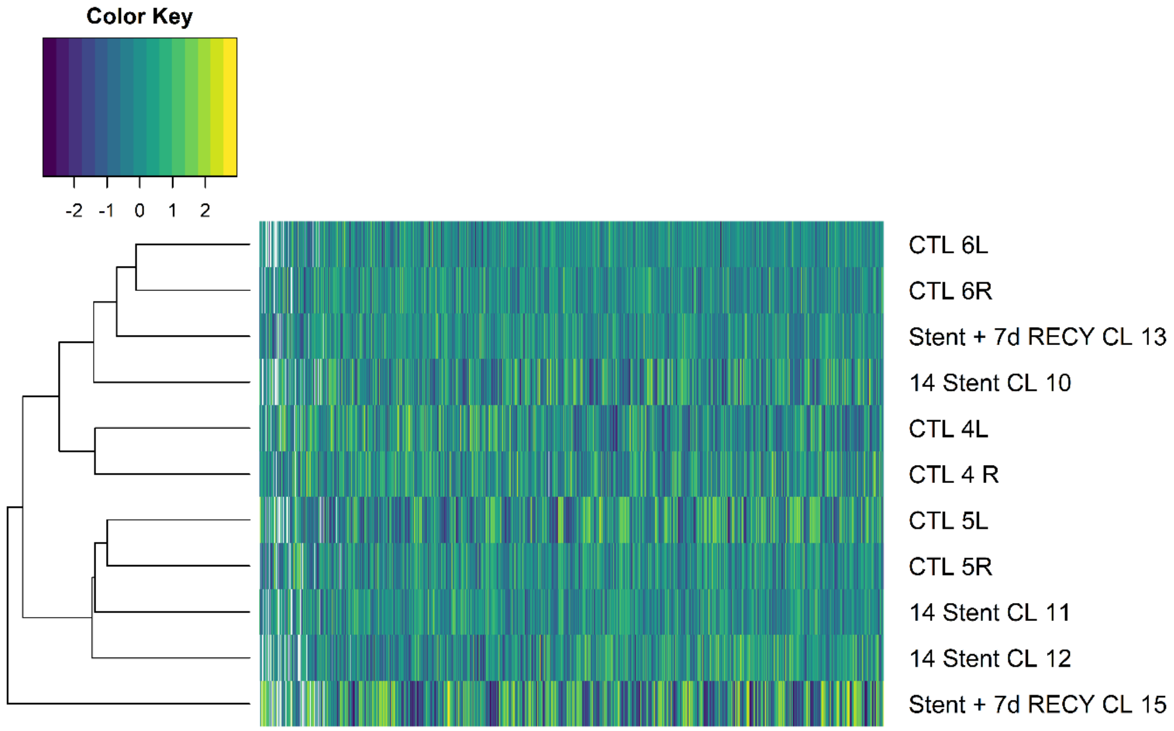

Figure S 3: Heatmap of gene expression of contralateral ureters of pigs that were stented for 14 days (14d Stent CL) or 14 days followed by 7 days of recovery (Stent + 7d RECY CL) and control ureters (CTL). Genes with more than 5 or more missing values were deleted (n=11029)

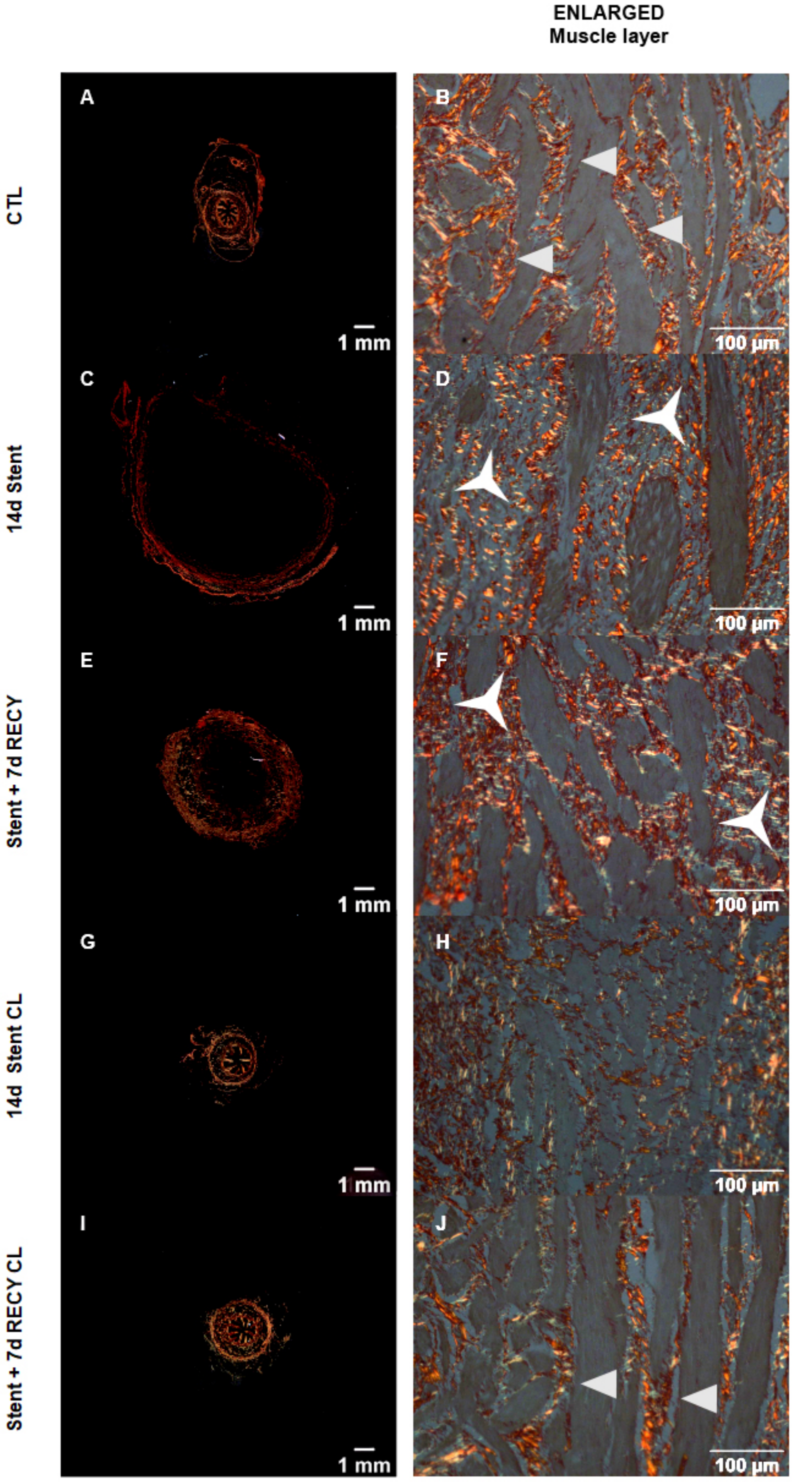

Figure S 4: Qualitative evaluation of fibrotic changes in stented ureters (A-J). (A, B) Regular collagen distribution in control ureters (CTL), (C, D) ureters stented for 14 days (14d Stent) and (E, F) 14 days followed by stent removal and recovery for 7 days (Stent + 7d RECY) and (G-J) their unstented contralateral ureters (14d Stent CL and STENT + 7D RECY CL). High magnification images of regular collagen distribution in the muscle layer (error heads in B), fibrosis (star) in 14d Stent (D) and Stent + 7d RECY (F). Low and high magnification images of Sirius red stains in polarized light.

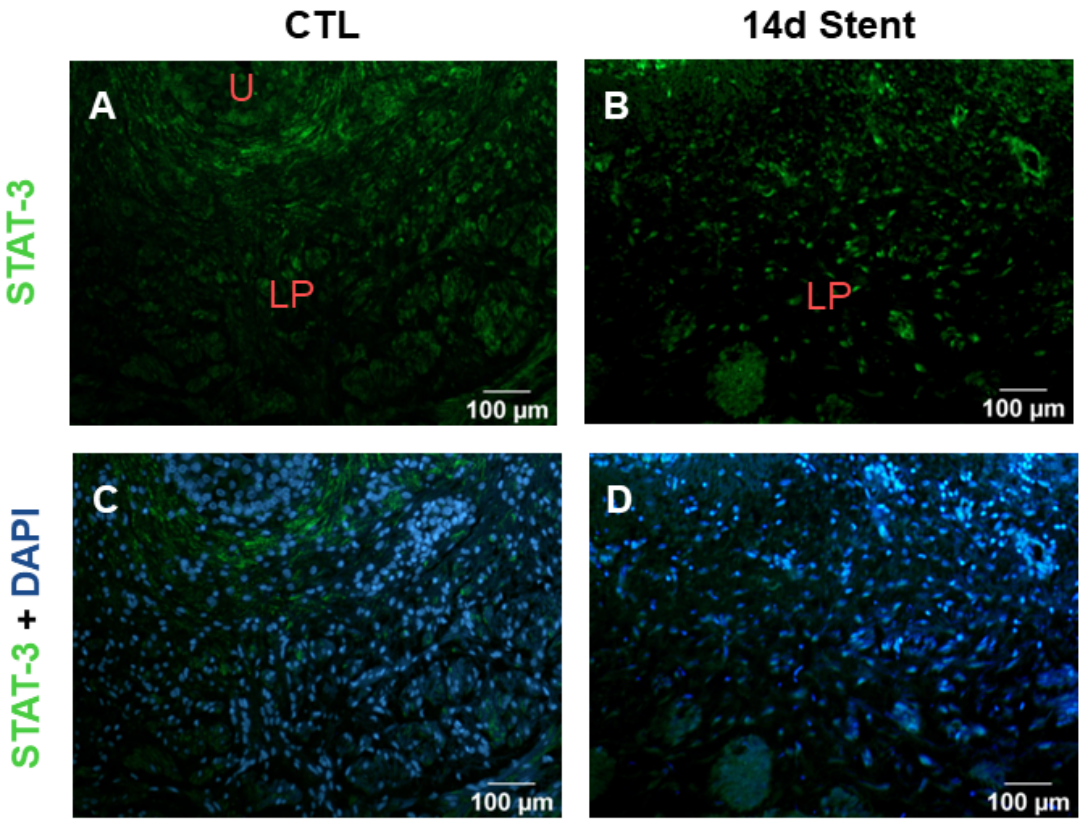

Figure S 5: Immunoflourescent staining of STAT3 in ureters. A and C shows staining for transcriptions factor STAT3 in control ureters (CTL), and B and D shows staining for STAT3 in ureters stented for 14 d (14d Stent). B and D showing positive nuclear staining for STAT3 in the lamina propria of stented ureters. U (Urothelium), LP (Lamina propria).

Table S 1: ***Histological grading system for inflammation and fibrosis in porcine ureters***

	0	1	2	3
**Urothelial integrity**	4 to 6 layers of cells, forming a star-shaped lumen	Same height, discontinuous, small vacuoles	Reduced height, small vacuoles, discontinuous	Significantly destructed, large vacuoles
**Fibrosis – Lamina propria**	Thin circumscript layer of collagen, not infiltrating the muscle layer	Mild changes	Moderate changes	Severe changes
**Fibrosis – Muscle layer**	Connective tissue between fibres barley observable in the muscle layer	Mild changes	Moderate changes	Severe changes
**Fibrosis - Adventitia**	Thin layer of collagen coating the muscle layer; fine reticular network of collagen fibres within the adipose tissue	Mild changes	Moderate changes	Severe changes
**Leucocyte infiltration – Lamina propria**	No observation of mononuclear cells	Few mononuclear cells observed	Diffuse infiltration of mononuclear cells	Clusters of mononuclear cells
**Leucocyte infiltration – Adventitia**	No observation of mononuclear cells	Few mononuclear cells observed	Diffuse infiltration of mononuclear cells	Clusters of mononuclear cells
**Hyperemia – Lamina propria**	Few observable blood vessels	Few observable blood vessels with increased diameter	Several observable blood vessels with increased diameter	Several observable blood vessels with increased diameter, erythrocytes in the tissue
**Hyperemia – Adventitia**	Few observable blood vessels	Few observable blood vessels with increased diameter	Several observable blood vessels with increased diameter	Several observable blood vessels with increased diameter, erythrocytes in the tissue

Table S 2: List showing proteins differentially expressed between 14d stented and control ureters. Significance was defined as a twofold change and p-value < 0.05.

Accession	Description	Gene Name	Fold change	p-value	Expression
P55944	Metallothionein-3	MT3	6.16	0.025587	upregulated
O79880	NADH-ubiquinone oxidoreductase chain 3	MT-ND3	5.7	0.020978	upregulated
F1SK41	Amino acid transporter	SLC1A1	4.62	0.001347	upregulated
Q8SPS7	Haptoglobin	HP	4.61	0.023727	upregulated
A0A287AIH1	4-hydroxyphenylpyruvate dioxygenase	HPD	4.48	0.000162	upregulated
F1SAR7	Uncharacterized protein	CLMN	4.22	0.008995	upregulated
F1SB19	Uncharacterized protein	PRPSAP2	4.11	0.011276	upregulated
F1RU54	Mitochondrial ribosomal protein L11	MRPL11	3.9	0.006033	upregulated
A0A287A3X9	6-phosphofructo-2-kinase/fructose-2,6-biphosphatase 2	PFKFB2	3.86	0.007696	upregulated
A0A287BG85	Collagen type XXVIII alpha 1 chain	COL28A1	3.83	0.000861	upregulated
F1SQG4	SECIS binding protein 2 like	SECISBP2L	3.18	0.000007	upregulated
F1SGG6	Keratin 5	KRT5	3.13	0.001043	upregulated
A0A287ASW4	Uncharacterized protein	Uncharacterized	3.07	0.016306	upregulated
A5A766	glycoprotein NMB	GPNMB	2.99	0.000049	upregulated
A0A286ZQ00	C-C motif chemokine	LOC100515857	2.92	0.000236	upregulated
F1SBY8	Uncharacterized protein	CROT	2.91	0.010673	upregulated
I3LM79	Dipeptidyl peptidase 9	DPP9	2.88	0.000135	upregulated
I3LEB7	Uncharacterized protein	PODN	2.87	0.012093	upregulated
A0A286ZWQ4	Chromosome 1 open reading frame 174	C1orf174	2.82	0.035859	upregulated
A0A287BDA0	Translocase of inner mitochondrial membrane 21	TIMM21	2.72	0.044233	upregulated
F1SDC2	Acid phosphatase 6, lysophosphatidic	ACP6	2.71	0.035329	upregulated
I3LNS9	Alpha-L-fucosidase	FUCA1	2.71	0.000049	upregulated
A0A286ZVC0	Tetratricopeptide repeat domain 7A	TTC7A	2.71	0.000008	upregulated
F1SFW4	Integrator complex subunit 3	INTS3	2.69	0.016902	upregulated
F1SGD2	Plakophilin 2	PKP2	2.67	0.000321	upregulated
Q9BDC3	p27Kip1	p27Kip1	2.65	0.000074	upregulated
P14287	Osteopontin	SPP1	2.65	0.000296	upregulated
F1RNU6	Uncharacterized protein	Uncharacterized	2.63	0.046245	upregulated
F1SKT8	Peptidylprolyl isomerase domain and WD repeat-containing 1	PPWD1	2.58	0.002518	upregulated
A0A287A6Z8	LLGL1, scribble cell polarity complex component	LLGL1	2.55	0.00008	upregulated
Q95274	Thymosin beta-4	TMSB4	2.54	0.011691	upregulated
I3LRU8	DEAH-box helicase 36	DHX36	2.54	0.030958	upregulated
A0A287B7S0	CD74 molecule	CD74	2.45	0.010333	upregulated
A0A287AAI1	C-type lectin domain containing 11A	CLEC11A	2.45	0.048823	upregulated
A0A287A9W0	Translocating chain-associated membrane protein	TRAM2	2.42	0.045527	upregulated
B3SP85	Gamma-interferon-inducible-lysosomal thiol reductase	IFI30	2.4	0.014581	upregulated
A0A287ARB4	Myosin VC	MYO5C	2.33	0.035872	upregulated
I3LKV0	Uncharacterized protein	SLIRP	2.33	0.013335	upregulated
A0A287AD55	DNA helicase	MCM2	2.29	0.008597	upregulated

A0A075B7I9	Uncharacterized protein	Uncharacterized	2.28	0.010573	upregulated
A0A287BQ22	O-6-methylguanine-DNA methyltransferase	MGMT	2.24	0.000978	upregulated
I3L728	Uncharacterized protein	Uncharacterized	2.19	0.036262	upregulated
A0A287ASZ4	Enah/Vasp-like	EVL	2.19	0.010989	upregulated
Q95JC8	Arginase-1	ARG1	2.19	0.030694	upregulated
A0A286ZN56	Uncharacterized protein	SEC62	2.17	0.02267	upregulated
F1SDG0	Uncharacterized protein	SF3B4	2.15	0.007124	upregulated
A0A286ZNS9	Ribosome production factor 2 homolog	RPF2	2.15	0.007627	upregulated
A0A287B3A4	Spectrin beta chain	SPTBN2	2.14	0.000001	upregulated
F1RSJ2	Uncharacterized protein	TRAPPC9	2.12	0.006257	upregulated
F1RPB1	Acyl-CoA synthetase medium chain family member 3	ACSM3	2.1	0.000166	upregulated
D3K5N3	Midkine	MDK	2.09	0.033039	upregulated
A0A287B0N7	Nucleolar protein 3	NOL3	2.09	0.008646	upregulated
Q06AU4	Ras-related protein Rab-34	RAB34	2.07	0.037105	upregulated
P00795	Cathepsin D	CTSD	2.06	0.039163	upregulated
A0A287AF99	Glutamyl-tRNA(Gln) amidotransferase subunit C, mitochondrial	GATC	2.06	0.044549	upregulated
A0A286ZID7	Transmembrane protein 41B	TMEM41B	2.06	0.002731	upregulated
F1SIC2	Uncharacterized protein	ACP2	2.05	0.002387	upregulated
A0A287B770	Serine and arginine rich splicing factor 10	SRSF10	2.04	0.019906	upregulated
F1RVV7	Cerebellar degeneration related protein 2 like	CDR2L	2.04	0.014015	upregulated
I3LA32	Uncharacterized protein	MINDY2	2.03	0.010377	upregulated
P80220	TSC22 domain family protein 3	TSC22D3	2.01	0.010191	upregulated
F1SGH0	Protein tyrosine phosphatase, receptor type G	PTPRG	2	0.043435	upregulated
F1RRW5	Angiotensin-converting enzyme	ACE	0.5	0.029883	downregulated
A0A286ZTC7	Anion exchange protein	SLC4A4	0.5	0.009279	downregulated
I3LJ48	Enoyl-CoA hydratase and 3-hydroxyacyl CoA dehydrogenase	EHHADH	0.49	0.002564	downregulated
A0A287BMY4	Dimethylglycine dehydrogenase	DMGDH	0.49	0.032537	downregulated
F1SEN2	Glutamate dehydrogenase 1, mitochondrial	GLUD1	0.49	0.001971	downregulated
A0A287B2W0	Uncharacterized protein	ALDH4A1	0.49	0.0301	downregulated
F1S8X9	Uncharacterized protein	HOGA1	0.49	0.02846	downregulated
A0A286ZID9	Uncharacterized protein	LOC110256481	0.49	0.006968	downregulated
A0A287AIJ3	Glutathione peroxidase	GPX3	0.49	0.019163	downregulated
A0A287A1E1	Uncharacterized protein	TRIAP1	0.49	0.001885	downregulated
A0A287BM32	Nucleoporin 43	NUP43	0.49	0.001073	downregulated
A5GZW8	Succinate dehydrogenase [ubiquinone] cytochrome b small subunit, mitochondrial	SDHD	0.48	0.004808	downregulated
F1S0C3	LYR motif containing 2	LYRM2	0.48	0.02238	downregulated
A0A287AT39	Acetyl-coenzyme A synthetase	ACSS1	0.47	0.000094	downregulated
F1RWY7	Uncharacterized protein	BPHL	0.47	0.000646	downregulated
A0A286ZIR9	Aminoadipate aminotransferase	AADAT	0.47	0.010545	downregulated
A1YIZ1	Selenide, water dikinase 2	SEPHS2	0.46	0.002826	downregulated
F1S7C8	Glutathione S-transferase alpha 4	GSTA4	0.46	0.031696	downregulated

F1RNC8	Uncharacterized protein	SLC13A2	0.46	0.026586	downregulated
F1S3H1	Aldehyde dehydrogenase 6 family member A1	ALDH6A1	0.45	0.043802	downregulated
A0A287ANI6	3-hydroxy-3-methylglutaryl-CoA lyase	HMGCL	0.45	0.009441	downregulated
A0A287BLI4	Pleckstrin homology domain containing A5	PLEKHA5	0.45	0.00025	downregulated
F6PS90	NAD-dependent protein deacetylase	SIRT3	0.45	0.039164	downregulated
A0A287BMA5	Uncharacterized protein	Uncharacterized	0.45	0.010951	downregulated
A0A286ZRI8	Arylsulfatase B	ARSB	0.45	0.03029	downregulated
F1RYP5	CWC22 spliceosome associated protein homolog	CWC22	0.44	0.009006	downregulated
F1S9I9	Mitochondrial amidoxime reducing component 2	44622	0.43	0.003124	downregulated
F1S5K2	Uncharacterized protein	SLC3A1	0.43	0.003204	downregulated
F1RUR0	UDP-glucuronosyltransferase	UGT2B31	0.43	0.001442	downregulated
P81649	Ribonuclease K3	RNASE6	0.43	0.016503	downregulated
F1RMV8	Cell adhesion molecule 4	CADM4	0.43	0.034121	downregulated
P41367	Medium-chain specific acyl-CoA dehydrogenase, mitochondrial	ACADM	0.42	0.015511	downregulated
F1SLE5	ATPase H+ transporting V1 subunit B1	ATP6V1B1	0.42	0.012533	downregulated
Q95333	Xaa-Pro aminopeptidase 2	XPNPEP2	0.42	0.023004	downregulated
Q02038	Neurolysin, mitochondrial	NLN	0.42	0.009492	downregulated
I3LK75	Nicotinate-nucleotide pyrophosphorylase [carboxylating]	QPRT	0.42	0.013019	downregulated
F1S0R9	Sarcosine dehydrogenase	SARDH	0.41	0.002468	downregulated
B8XH67	Na(+)/H(+) exchange regulatory cofactor NHE-RF	SLC9A3R1	0.41	0.000156	downregulated
I3LVF1	Hydroxyacid oxidase 1	HAO1	0.41	0.001145	downregulated
A0A287ATM3	Sulfate anion transporter 1	SLC26A1	0.41	0.003349	downregulated
F1SAI9	Solute carrier family 37 member 4	SLC37A4	0.41	0.000438	downregulated
I3LLU2	Solute carrier family 43 member 2	SLC43A2	0.41	0.000143	downregulated
A0A287B6X3	Lambda-crystallin homolog	CRYL1	0.4	0.000045	downregulated
P00371	D-amino-acid oxidase	DAO	0.4	0.031716	downregulated
F1SK21	Ammonium transporter Rh type C	RHCG	0.4	0.025239	downregulated
F1RTA3	Folate gamma-glutamyl hydrolase	GGH	0.4	0.000861	downregulated
Q35916	Cytochrome c oxidase subunit 3	MT-CO3	0.4	0.010083	downregulated
P37111	Aminoacylase-1	ACY1	0.39	0.000401	downregulated
F1SKY2	Nitrilase family member 2	NIT2	0.39	0.00299	downregulated
A0A287AAP0	Uncharacterized protein	LOC100522130	0.39	0.000847	downregulated
F1SH89	Choline dehydrogenase	CHDH	0.39	0.000424	downregulated
I3LQ57	Phosphotriesterase related	PTER	0.39	0.000924	downregulated
F1S451	Lactate dehydrogenase D	LDHD	0.39	0.000256	downregulated
A0A2C9F343	Lysozyme	LYZ	0.39	0.008634	downregulated
A0A287BK91	Tetratricopeptide repeat domain 7B	TTC7B	0.39	0.000199	downregulated
A0A287A9U9	Solute carrier family 22 member 1	SLC22A1	0.38	0.018045	downregulated
F1SPI0	Uncharacterized protein	CHCHD4	0.38	0.002712	downregulated
P00636	Fructose-1,6-bisphosphatase 1	FBP1	0.37	0.001701	downregulated
I3LEH4	Amine oxidase	MAOB	0.37	0.018676	downregulated

F1RJH8	Argininosuccinate lyase	ASL	0.37	0.000001	downregulated
Q9TRC7	Amiloride-sensitive amine oxidase [copper-containing]	AOC1	0.37	0.009688	downregulated
F1SUP1	Uncharacterized protein	AKR7A2	0.37	0.000127	downregulated
A0A286ZJA4	Uncharacterized protein	CYP2C36	0.37	0.021292	downregulated
F1SD56	MISP family member 3	MISP3	0.37	0.000828	downregulated
I3LQD0	Uncharacterized protein	CALML4	0.37	0.01499	downregulated
F1RPB0	Uncharacterized protein	ACSM2B	0.36	0.000768	downregulated
P79431	Metallothionein-1E	MT1E	0.36	0.006847	downregulated
A0A287ADL4	Polymeric immunoglobulin receptor	PIGR	0.36	0.009647	downregulated
A0A286ZTA4	Fructose-bisphosphate aldolase	ALDOB	0.35	0.004061	downregulated
I3LGB4	Tubulointerstitial nephritis antigen	TINAG	0.35	0.00158	downregulated
I3LEZ7	Histone deacetylase 6	HDAC6	0.35	0.000024	downregulated
F1RWI8	Secernin 2	SCRN2	0.35	0.000614	downregulated
A0A287BB52	Inositol oxygenase	MIOX	0.35	0.000182	downregulated
A0A287BQ20	Uncharacterized protein	Uncharacterized	0.35	0.008795	downregulated
F1RWC3	Cubilin	CUBN	0.34	0.00011	downregulated
P22411	Dipeptidyl peptidase 4	DPP4	0.34	0.005738	downregulated
I3LTZ8	Phenazine biosynthesis like protein domain containing	PBLD	0.34	0.00621	downregulated
F1SIX9	Glycerate kinase	GLYCTK	0.34	0.003543	downregulated
F1RMF6	Uncharacterized protein	LOC100517803	0.34	0.005358	downregulated
D0VWV4	Succinate dehydrogenase cytochrome b560 subunit, mitochondrial	SDHC	0.34	0.000006	downregulated
P80147	4-aminobutyrate aminotransferase, mitochondrial	ABAT	0.33	0.041918	downregulated
F1SND2	Uncharacterized protein	AGXT2	0.33	0.005261	downregulated
I3LSX8	Cadherin 16	CDH16	0.33	0.000022	downregulated
A0A287AT21	Sorbitol dehydrogenase	SORD	0.33	0.001513	downregulated
I3LQA1	Aldehyde dehydrogenase 8 family member A1	ALDH8A1	0.33	0.000069	downregulated
A0A287BS58	Sarcoglycan epsilon	SGCE	0.33	0.003045	downregulated
P00172	Cytochrome b5	CYB5A	0.33	0.00235	downregulated
I3LSY0	Acyl-CoA synthetase medium chain family member 4	ACSM4	0.32	0.00107	downregulated
A0A287AES5	Phosphoenolpyruvate carboxykinase 2, mitochondrial	PCK2	0.31	0.00004	downregulated
F1RL81	Hydroxysteroid 17-beta dehydrogenase 14	HSD17B14	0.31	0.000212	downregulated
A0A287A2X5	Gluconokinase	IDNK	0.31	0.005877	downregulated
C0HL13	Low-density lipoprotein receptor-related protein 2	LRP2	0.3	0.000122	downregulated
A0A287BTN2	Succinyl-CoA:glutarate-CoA transferase	SUGCT	0.3	0.005814	downregulated
A0A287AKZ9	V-type proton ATPase subunit G	ATP6V1G1	0.3	0.000006	downregulated
F1REY0	Uncharacterized protein	VPS37B	0.3	0.00196	downregulated
I3LJJ8	PDZ domain containing 1	PDZK1	0.29	0.006985	downregulated
P50441	Glycine amidinotransferase, mitochondrial	GATM	0.28	0.000059	downregulated
I3L719	Dipeptidase	DPEP1	0.28	0.000143	downregulated
F1S1E7	Dihydropyrimidinase	DPYS	0.28	0.000206	downregulated
B3VFB6	Calbindin 1	CALB1	0.28	0.000058	downregulated

F1S971	Centrosomal protein 170B	CEP170B	0.28	0.014689	downregulated
I3LFP5	PDZK1-interacting protein 1	PDZK1IP1	0.28	0.015207	downregulated
F1SBB3	Laminin subunit alpha 3	LAMA3	0.27	0.02607	downregulated
A0A286ZXK0	Uncharacterized protein	PARD6B	0.27	0.02884	downregulated
F1SQH7	Solute carrier family 27 (Fatty acid transporter) member 2	SLC27A2	0.26	0.004386	downregulated
A0A287A7R1	N-acetyltransferase 8 (putative)	NAT8	0.26	0.012175	downregulated
F1SRG4	Cytochrome P450 2D6	CYP2D25	0.25	0.035691	downregulated
A0A287AZ62	Uncharacterized protein	CYP2C33	0.23	0.000357	downregulated
A0A287AXR4	Arylacetamide deacetylase	AADAC	0.23	0	downregulated
A0A287ABJ0	Uncharacterized protein	BHMT2	0.22	0.000005	downregulated
F1SNP0	Uncharacterized protein	COL4A3	0.22	0.014172	downregulated
F1SJM4	Neprilysin	MME	0.21	0.000015	downregulated
A0A287BCM4	Uncharacterized protein	Uncharacterized	0.21	0.000848	downregulated
F1RL41	Beta-ureidopropionase 1	UPB1	0.2	0.000058	downregulated
B5KFA0	Aromatic-L-amino-acid decarboxylase	AADC	0.19	0.000837	downregulated
A0A287A9G4	Argininosuccinate synthase 1	ASS1	0.18	0.000007	downregulated
E7D6R2	Betaine homocysteine methyltransferase	bhmt	0.18	0.000231	downregulated
Q29261	Villin-1	VIL1	0.17	0.000024	downregulated
F1SUU6	Agmatinase	AGMAT	0.17	0.000028	downregulated
I3LKT6	Solute carrier family 5 member 2	SLC5A2	0.17	0.000926	downregulated
A0A287AT23	Aquaporin-1	AQP1	0.17	0.000232	downregulated
F1S1C3	Uncharacterized protein	CA4	0.16	0.000018	downregulated
I3LCS3	Solute carrier family 23 member 1	SLC23A1	0.12	0.015013	downregulated
A0A286ZM75	Uncharacterized protein	LOC102165484	0.12	0.000002	downregulated
P79377	Metallothionein-1D	MT1D	0.11	0.004581	downregulated
Q70BM6	Solute carrier family 22 member 8	SLC22A8	0.09	0	downregulated
F1SAM6	FXYD domain-containing ion transport regulator	FXYD2	0.06	0.000038	downregulated
